# Supplementary material for: The Synthesis of Manganese Hydroxide Nanowire Arrays for a High-Performance Zinc-Ion Battery
Source: Nanomaterials (Basel). 2022 Jul 22;12(15):2514. doi: 10.3390/nano12152514 (PMC9331603; doi:10.3390/nano12152514)
Supplement: Supplementary file 1 [file nanomaterials-12-02514-s001.zip › nanomaterials-1830007-supplementary.pdf]

Supporting Information

Synthesis of Manganese Hydroxide Nanowire Arrays for High-Performance Zinc-ion Battery

Jiangfeng Gong [1,*], Bingxin Zhu [1], Zhupeng Zhang [1], Yuanyuan Xiang [1], Chunmei Tang [1,*], Qingping Ding [2] and Xiang Wu [3,*]

1 College of Science, Department of Physics, Hohai University, Nanjing 210098, China;

2 Ames Laboratory and Department of Physics and Astronomy, Iowa State University, Ames, Iowa 50011, USA

3 School of Materials Science and Engineering, Shenyang University of Technology, Shenyang 110870, China

*Correspondence: jfgong@hhu.edu.cn (J.G.); cmtang@hhu.edu.cn (C.T.); wuxiang05@sut.edu.cn (X.W.)

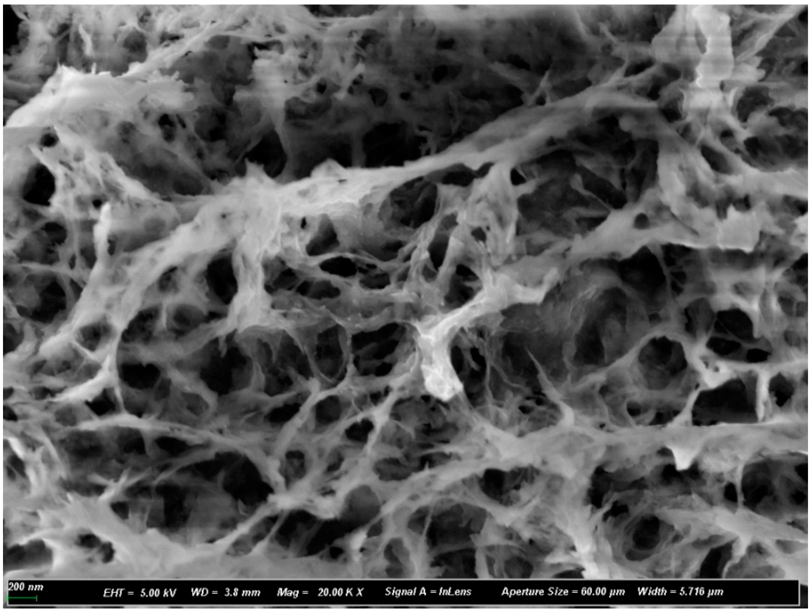

Figure S1. SEM images of the Mn(OH)2 after long-life cycle.

Supporting Notes:

Calculation details of the Galvanostatic Intermittent Titration Technique (GITT):

GITT analysis was applied to determine the Zn^{2+} ion diffusion coefficients ($D_{Zn}{}^{2+}$, cm^2 s^{-1}), following the methodology described in refs. In GITT analysis, the transient voltage that is generated due to the application of a current pulse is monitored as a function of time. Before the GITT measurement, the assembled cell was first discharged/charged at 0.3 A g^{-1} for 30 cycles to obtain a stable state. Subsequently, a galvanostatic pulse (charge or discharge) of 3000 sec at a current density of 50 mA g^{-1} was followed by 60 sec at open circuit step to allow relaxation back to equilibrium. In general, pulse times range from 10 minutes to several hours, depending on the material and its kinetics. Electrode materials with fast reaction rates, i.e. with high diffusion coefficients require shorter time pulse times so as to avoid parasitic side reactions during the hold, once the primary redox reaction is complete. Because of the fast charge behavior of the PANI, 3000 sec pulse duration and relaxation time ware chosen. This was repeatedly applied until the discharge (charge) voltage reached 0.3 V (1.8V) vs. Zn.

$$D_S = \frac{4}{\tau\pi}\left(\frac{n_M V_M}{S}\right)^2\left(\frac{dE_s}{dE_\tau}\right)^2$$

Where τ is the constant current pulse duration (3000 sec); n_M and V_M are the moles (mol) of PANI and molar volume (cm^3 mol^{-1}), respectively. S is the electrode electrolyte interface area (cm^2) taken as the geometric area of the electrode; dE_s and dE_τ are the change in the steady state voltage and overall cell voltage after the application of a current pulse in a single step GITT experiment.

Table S1. Diffusion coefficient of Zn^{2+} in referenced cathode materials.

Active materials	Electrolyte	Discharge $D_{Zn^{2+}}$ (cm^{-2} s^{-1})	Charge $D_{Zn^{2+}}$ (cm^{-2} s^{-1})	Reference
α-MnO_2 microspheres	2M $ZnSO_4$ 0.1M $MnSO_4$	10^{-9}~10^{-12}	10^{-8}~10^{-11}	1
Birnessite MnO_2 Nanobelts	2 M $ZnSO_4$ 0.2 M $MnSO_4$	8.18 x 10^{-14}		2
δ-MnO_2	1M $ZnSO_4$ 0.1M $MnSO_4$	1.3 x 10^{-12} ~ 8.9 x 10^{-14}		3
ε-MnO_2	2M $ZnSO_4$ 0.5M $MnSO_4$	2.96 × 10^{-14}		4
Fe/α-MnO_2	2M $ZnSO_4$ 0.1M $MnSO_4$	10^{-10}~10^{-14}		5
MnO_2 NWs	3M $Zn(CF_3SO_3)_2$ 0.1M $Mn(CF_3SO_3)_2$	3.1 × 10^{-13}		6
V_2O_3@C	3M $Zn(CF_3SO_3)_2$	1.6 ×10^{-14}~8.6 ×10^{-10}	9.6 ×10^{-11}~3.6 ×10^{-10}	7
PANI-VOH	3M $Zn(TfO)_2$ 6M LiTFSI	5.6 ×10^{-16}~3.6 ×10^{-13}	6.8×10^{-14}~1.2×10^{-13}	8
δ-$Ni_{0.25}V_2O_5 \cdot nH_2O$	3 M $ZnSO_4$	10^{-10}		9
V_2O_5nanowires	3M $ZnSO_4$	1.01 × 10^{-12}~8.40 × 10^{-10}		10
V_2O_5/CNTs composite film	3 M $Zn(CF_3SO_3)_2$	~10^{-9}		11
PANI film	2 M $Zn(CF_3SO_3)_2$	6.25 × 10^{-9}~7.82 × 10^{-8}	7.69×10^{-10}~1.81× 10^{-7}	12
V_2CT_x MXene	2 M $Zn(CF_3SO_3)_2$	3.7 × 10^{-13}		13
PC@MFO	2 M $ZnSO_4$	10^{-14}~10^{-10}		14
Manganese Hydroxide Nanowire Arrays	2 M $Zn(CF_3SO_3)_2$	1.0×10^{-9}~2.7×10^{-11}	4.5×10^{-8}~1.0×10^{-9}	**This work**

Reference:

1. Wu, Y.; Tao, Y.; Zhang, X.; Zhang, K.; Chen, S.; Liu, Y.; Ding, Y.; Cai, M.; Liu, X.; Dai, S., Self-assembled alpha-MnO_2 urchin-like microspheres as a high-performance cathode for aqueous Zn-ion batteries. *Sci. China Mater.* **2020,** *63*, 1196-1204.
2. Wang, Y.; Ye, F.; Wu, Z.; Jiang, L.; Zhang, L.; Hu, L., Macroporous, Freestanding Birnessite $H_{0.08}MnO_2$ center dot 0.7H_2O Nanobelts/Carbon Nanotube Membranes for Wearable Zinc-Ion Batteries with Superior Rate Capability and Cyclability. *ACS Appl. Energy Mater.* **2021,** *4*, 4138-4149.
3. Guo, C.; Liu, H.; Li, J.; Hou, Z.; Liang, J.; Zhou, J.; Zhu, Y.; Qian, Y., Ultrathin delta-MnO_2 nanosheets as cathode for aqueous rechargeable zinc ion battery. *Electrochim. Acta* **2019,** *304*, 370-377.
4. Zhang, Y.; Liu, Y.; Liu, Z.; Wu, X.; Wen, Y.; Chen, H.; Ni, X.; Liu, G.; Huang, J.; Peng, S., MnO_2 cathode materials with the improved stability via nitrogen doping for aqueous zinc-ion batteries. *J. Energy Chem.* **2022,** *64*, 23-32.
5. Xu, J.-W.; Gao, Q.-L.; Xia, Y.-M.; Lin, X.-S.; Liu, W.-L.; Ren, M.-M.; Kong, F.-G.; Wang, S.-J.; Lin, C., High-performance reversible aqueous zinc-ion battery based on iron-doped alpha-manganese dioxide coated by polypyrrole. *J. Colloid Interface Sci.* **2021,** *598*, 419-429.
6. Lian, S.; Sun, C.; Xu, W.; Huo, W.; Luo, Y.; Zhao, K.; Yao, G.; Xu, W.; Zhang, Y.; Li, Z.; Yu, K.; Zhao, H.; Cheng, H.; Zhang, J.; Mai, L., Built-in oriented electric field facilitating durable Zn-MnO_2 battery. *Nano Energy* **2019,** *62*, 79-84.
7. Liu, Y.; Liu, Y.; Wu, X.; Cho, Y.-R. Enhanced Electrochemical Performance of Zn/VO_x Batteries by a Carbon-Encapsulation Strategy. *ACS Appl. Mater. Interfaces* **2022,** *14*, 11654-11662.
8. Wang, M.; Zhang, J.; Zhang, L.; Li, J.; Wang, W.; Yang, Z.; Zhang, L.; Wang, Y.; Chen, J.; Huang, Y.; Mitlin, D.; Li, X., Graphene-like Vanadium Oxygen Hydrate (VOH) Nanosheets Intercalated and Exfoliated by Polyaniline (PANI) for Aqueous Zinc-Ion Batteries (ZIBs). *ACS Appl. Mater. Interfaces* **2020,** *12*, 31564-31574.
9. Li, J.; McColl, K.; Lu, X.; Sathasivam, S.; Dong, H.; Kang, L.; Li, Z.; Zhao, S.; Kafizas, A. G.; Wang, R.; Brett, D. J. L.; Shearing, P. R.; Cora, F.; He, G.; Carmalt, C. J.; Parkin, I. P., Multi-Scale Investigations of delta-$Ni_{0.25}V_2O_5$ center dot nH_2O Cathode Materials in Aqueous Zinc-Ion Batteries. *Adv. Energy Mater.* **2020,** *10*, 2000058.
10. Mao, F.; Li, Y.; Zou, Z.; Huang, B.; Yang, J.; Yao, J., Zn^{2+} storage performance and structural change of orthorhombic V_2O_5 nanowires as the cathode material for rechargeable aqueous zinc-ion batteries. *Electrochim. Acta* **2021,** *397*, 139255.
11. Liu, X.; Ma, L.; Du, Y.; Lu, Q.; Yang, A.; Wang, X., Vanadium Pentoxide Nanofibers/Carbon Nanotubes Hybrid Film for High-Performance Aqueous Zinc-Ion Batteries. *Nanomater.* **2021,** *11*, 1054.
12. Gong, J.; Li, H.; Zhang, K.; Zhang, Z.; Cao, J.; Shao, Z.; Tang, C.; Fu, S.; Wang, Q.; Wu, X., Zinc-Ion Storage Mechanism of Polyaniline for Rechargeable Aqueous Zinc-Ion Batteries. *Nanomater.* **2022,** *12*, 1438.
13. Sha, D.; Lu, C.; He, W.; Ding, J.; Zhang, H.; Bao, Z.; Cao, X.; Fan, J.; Dou, Y.; Pan, L.; Sun, Z., Surface Selenization Strategy for V_2CT_x MXene toward Superior Zn-Ion Storage. *ACS Nano* **2022,** *16*, 2711-2720.
14. Liu, X.; Shen, X.; Chen, T.; Xu, Q., The spinel $MnFe_2O_4$ grown in biomass-derived porous carbons materials for high-performance cathode materials of aqueous zinc-ion batteries. *J. Alloys Compd.* **2022,** *904*, 164002.
